# Say what you want, I’m not listening! A conversational self-reflection robot that does not parse user speech

**DOI:** 10.1515/icom-2022-0047

**Published:** 2023-03-10

**Authors:** Adriana Lorena González, Denise Y. Geiskkovitch, James E. Young

**Affiliations:** Department of Computer Science, University of Manitoba, Winnipeg, Canada; Department of Computing and Software, McMaster University, Hamilton, Canada

**Keywords:** companion robots, conversational robot, domestic robots, robots in the wild, self-reflection therapy, social robotics

## Abstract

We present a conversational social robot behaviour design that draws from psychotherapy research to support individual self-reflection and wellbeing, without requiring the robot to parse or otherwise understand what the user is saying. This simplicity focused approached enabled us to intersect the well-being aims with privacy and simplicity, while achieving high robustness. We implemented a fully autonomous and standalone (not network enabled) prototype and conducted a proof-of-concept study as an initial step to test the feasibility of our behaviour design: whether people would successfully engage with our simple behaviour and could interact meaningfully with it. We deployed our robot unsupervised for 48 h into the homes of 14 participants. All participants engaged with self-reflection with the robot without reporting any interaction challenges or technical issues. This supports the feasibility of our specific behaviour design, as well as the general viability of our non-parsing simplicity approach to conversation, which we believe to be an exciting avenue for further exploration. Our results thus pave the way for further exploring how conversational behaviour designs like ours may support people living with loneliness.

## 1 Introduction

It’s good company. My daughter says I’m antisocial. I like to be alone, but I turn the robot on. I don’t feel so lonely – P11, exit interview

Social robots leverage people’s tendency to anthropomorphize, using animal or human-like techniques to enrich and simplify interaction [1]. In addition to basic communication (e.g., a smile to represent state), social robots can be designed to influence emotional well-being, to decrease loneliness [2], stress [3], or provide emotional support [4]; in general, such *socially assistive robots* are designed to aid people by way of social interaction [5]. Perhaps more than other technologies, social robots offer a physical and social presence more akin to collocated inter-personal interaction, providing opportunities for novel well-being support designs (e.g., see [6–8]). Following, we designed a novel social robot behavior, drawing from self-reflection and well-being research (e.g., see [9, 10]), that engages people to reflect critically on recent experiences. We further conducted an in-the-wild evaluation with a fully autonomous prototype, with positive results validating the interaction design and supporting longitudinal study of use patterns and impact on well-being.

Self-reflection is the process of evaluating one’s own thoughts, emotions, attitudes, and behaviors. It can support introspection and flexible thinking, enabling one to better understand situations and feelings [11, 12]. Related techniques such as a thought diary [13] or talking to others about one’s feelings [14, 15] can improve mood. Technology-mediated approaches are emerging such as digital social networks [16] or online automated “chatbots” [17] to support reflection, with some evidence that they can reduce feelings of loneliness [16, 17]. Similarly, social robots have been used as conversational partners for a range of applications, including well-being support (see, e.g., [18]).

**Figure 1:**
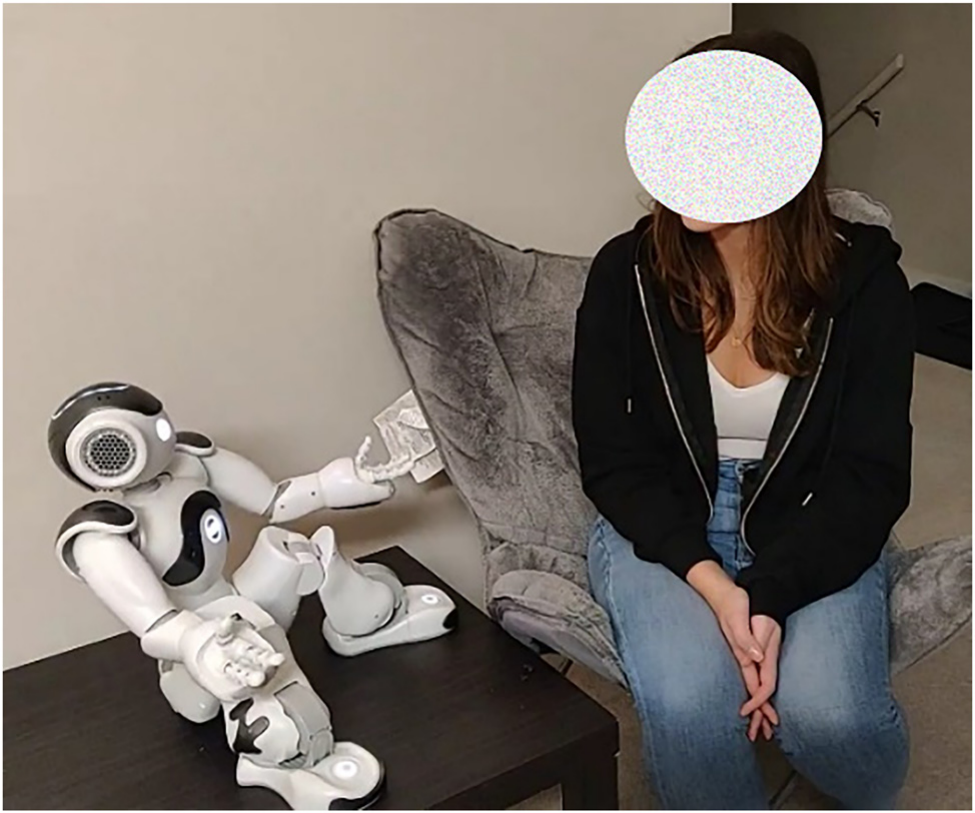
Our conversational robot design uses well timed simple questions and prompts to encourage people to self-reflect, aiming to support well-being without needing the robot to parse or understand what the user says. (Recreated interaction, participants were not recorded).

However, successful self-reflection agent deployment remains elusive for a range of reasons (e.g., see [19]) including the technical challenges of speech recognition and parsing [20] and overarching privacy concerns [21]. Within this context, we aimed to bypass many of these problems by removing the requirement for the robot to parse the content of user utterances – we propose that a conversational behavior design can provide many of the self-reflection benefits of conversational agents, even without recording or parsing what a person says. Our design process resulted in a novel social conversational robot behavior design that leverages anthropomorphism to support social engagement, has complete privacy to facilitate candid interaction, maintains interaction simplicity to support light-weight engagement and avoid distracting interaction, and is technically simple, resulting in a robust, offline interaction. Specifically, in our prototype users interact with a humanoid social robot (Figure 1) to talk about their daily life and feelings, to encourage self-reflection, while the robot provides well-timed prompts and body-language feedback.

We implemented a fully autonomous prototype of our design and conducted an initial evaluation to examine the practical and technical feasibility of our behavior and prototype, supporting early-stage iteration [22]. We deployed our prototype into the homes of 14 older adults for 48 h each; we selected this demographic as older adults are more likely than younger adults to live alone and experience loneliness [23, 24]. However, we did not target lonely people at this early stage of the research given the uncertainty about the interaction success of our prototype. Our results indicate that people successfully engaged in self-reflection with the robot, with no one reporting interaction errors or confusion; all participants noted the ease of use. People reported being comfortable talking with the robot even for sensitive topics, highlighting the success of our privacy-driven design. Most participants reported feeling that the self-reflection was helpful, and all reported that the prototype acted as a social catalyst to help them engage with others. Further, the robot operated fully autonomously without requiring assistance. However, over the course of our deployment people generally reported reducing their opinions of social robots as a result, despite the interaction success. Thus, these results support the interaction and technical feasibility of our non-parsing conversational approach, paving the way for exploring novel designs and the potential positive impact of designs like these on users’ well-being.

Our contribution is not technical, as our implementation was quite simple. Rather, we propose a novel design approach to conversational agents which challenges conversational agent designers to consider how user preparation and conversation timing may enable designs to reduce their technical requirements. With the study results providing initial success, this points to further explorations on conversational designs that could benefit from the added privacy and robustness benefits of not parsing user input, while balancing the need for interactive conversation depth.

## 2 Related work

Socially assistive robots have the potential to improve mental health and wellbeing, reducing loneliness, anxiety, and improve quality of life [25–27]. Such robots can serve in companionship roles, such chatting, telling jokes, or playing music (e.g., [18]). In care homes, even simple chatting or playing games increases engagement, and people enjoy having robots around [28–30]. However, these works generally target specific vulnerable populations (e.g. [2, 31, 32]), with less research for generally healthy people.

Although computerized conversational agents have long been designed to support well-being (e.g., even the early ELIZA chatbot was programmed to mimic a psychotherapist [33]), effective conversational agent uptake for well-being remains elusive [8]; research continues to explore novel designs and approaches (e.g., [34–40]). One thread for improving engagement has been to embed chat agents into embodied characters, for example, a virtual character for delivering cognitive behavior therapy or related approaches [8, 35, 36, 41]; this embodiment may elicit more engagement than a purely text interface [8]. Social robots have similarly been used for cognitive behavior therapy [42] and positive psychology [20, 37], or more generally designed to be companions to support well-being [7, 20, 43]. While some projects indicate positive well-being outcomes (e.g. [8, 35, 42]), overall results are mixed with reviews failing to find consistent benefits [34, 38], and user drop-out rates remain high [20].

Although virtual agents have been deployed into fully natural settings (e.g., [8, 28, 29, 41]), autonomous conversation robots that are able to hold complex conversations that span over several minutes and on diverse topics have rarely been studied in natural domestic settings (a recent exception being [43]). Longer term studies either use the Wizard of Oz technique [42], have experts monitor in real time (e.g., collocated, [28]; or with video [44]), or deploy as kiosks in shared environments [37, 45]. Perhaps this is because fully autonomous interactive assistive robots remain rare. Many prototypes rely on a real-time secret operator [46] to select operations or parse input (e.g. [18, 35]), greatly reducing deployment feasibility. More autonomous solutions still often require structured environments with expert supervision (e.g. [20, 45]), precluding domestic deployment. Such setups can diminish the validity of results (e.g., as with the Hawthorne effect [47]), as participants are interacting in highly controlled unnatural (e.g. [45]), or observed (e.g., via video [44]) settings. We designed one of the first fully autonomous conversational robots to be studied within people’s homes.

Autonomous natural dialog interaction remains elusive for interactive agents; both stable speech recognition and semantic parsing remain an ongoing challenge [20, 48]. As a result, a typical format is agent-led content delivery, where an agent monitors for expected utterances, keywords, or events and provides pre-scripted responses and dialog flow (e.g., [35, 37]). Others directly require users to select specific responses, for example, via a touch screen (e.g., [20, 49]). The result is often a simple interaction where users are primarily navigators of agent-provided content that is highly scripted and undynamic (e.g., see [18, 35, 37]). Machine learning is being used to improve both speech recognition and generation, although many systems still require a human in the loop to achieve satisfactory results (e.g. [42]), and results suffer from lack of interaction depth and poor generation quality [34, 49]. We aim to solve this problem through designing interaction that simultaneously increases the role of the human as the content creator, while reducing the need for the robot to parse human utterances.

Finally, many systems support well-being via simulating emotional connections with a user (e.g., [7, 40, 50]). This raises ethical questions about the roles of social agents and the reliance on the fundamental deception that presents agents as actual social beings [21, 40, 50]. Further, there are additional risks with relying on generated content in sensitive situations such as for well-being or health in general [21, 51], as the unintelligent robot may inadvertently say something harmful. Our work sidesteps these issues by minimizing the need for agent content generation or simulated friendship, thus enabling a designer to beforehand know with certainty what utterances a robot will make.

## 3 Interaction design

Given the early stage of the work we first developed a proof-of-concept prototype for the self-reflection approach, and then used it as a technology probe [52] to engage users within the context of use (see Section 4) to inform iteration on the prototype. This enables us to reflect on the design approach itself while leaving the inquiry on well-being outcomes for a later research stage.

### 3.1 Design goals and approach

We devised several design goals (in italics) that we detail below. First, the robot should provide *social engagement* and draw from *reflective-listening practices* to encourage and support the desired self-reflection behavior, based on the therapy model of the person as the content generator (through self-reflection) and the robot as the self-reflection facilitator. Second, to minimize required learning and reduce the barrier to engage the robot we aim for *interaction simplicity*. Further, given the sensitive nature of self-reflection we need to emphasize interaction *safety and privacy.* Finally, we focus on *implementation simplicity* to improve robustness, remove the need for remote monitoring, and enable fully autonomous in-home deployment. Below we detail how we approached each goal in our robot and interaction design.

#### 3.1.1 Reflective-listening practices

We drew from reflective listening approaches in therapy where the listener’s role (traditionally a therapist, here the robot) is to primarily provide discussion and reflection prompts and listen as the client talks [53, 54]. Thus, our aim was to have the robot encourage an individual to talk and self-reflect rather than the robot giving advice. The robot listener should be non-judgmental and accepting, to increase comfort [53, 54].

#### 3.1.2 Social engagement

To help interaction between the user and the robot feel more natural, we used a humanoid social robot platform and programmed it with active listening behaviors including tracking (and looking at) the user’s face and using gestures while talking. This further leverages the potential for increased engagement of robots in comparison to virtual agents (e.g., [7, 55]).

#### 3.1.3 Interaction simplicity

To avoid detracting from interaction and to encourage engagement, we aimed for as-simple-as-possible interaction that avoids complex features that may overwhelm users (e.g., as in [22]). As part of this, the robot design must be careful to avoid creating unrealistic user expectations and be transparent about its abilities [56], as inflated perceptions of robot ability can lead to interaction troubles and disappointment [57].

#### 3.1.4 Safety and privacy

Users need to feel comfortable disclosing sensitive information during self-reflection. We aimed for a fully autonomous design that does not require remote monitoring and does not record user utterances. To further increase the sense of safety and privacy we aimed for a fully offline solution, as any platform that relies on online infrastructure (e.g., for processing, [20, 37, 58]). May reduce a sense of privacy and restrict candid interaction. We also note that these designs must be transparent to users. Further, given the dangers of generating robot utterances (i.e., the increased potential to make harmful or offensive statements as a result of the lack of content oversight), we aimed for a design that does not require dialog generation, enabling the researchers to ensure the safety of robot utterances.

#### 3.1.5 Implementation simplicity

Unsupervised in-home deployment requires an implementation simplicity that avoids remote monitoring or operation (e.g., no Wizard-of-Oz techniques, [46]); this further helps achieve our safety and privacy goals. Our approach was to, whenever possible, replace a technical solution with a design solution that trades complex sensing for a user action. Seeking technically simple ways to realize social interaction goals can achieve the benefits of social robots while reducing the need for real time monitoring, control, or fragile behavior algorithms.

Overall, we aim for as-simple-as-possible interaction and implementation that meets our self-reflection interaction goals.

### 3.2 Prototype design

We designed, informally piloted (with team members), and iteratively revised our prototype while focusing on the above design goals. Earlier prototypes previously appeared as work-in-progress publications without deployment [59, 60]. As our platform, we settled on a humanoid robot given the potential to facilitate social engagement and natural conversation.

#### 3.2.1 In-home integration and engagement

The prototype is designed to be placed at a fixed location in a person’s home, where it sits passively, simplifying implementation by removing the need for localization or navigation algorithms. Further, instead of the robot choosing when to engage, which may require configuration or be error prone (e.g., misinterpret or ask at an awkward time), we leave the robot to sit passively – the user can engage the robot whenever they desire; this simplifies both interaction and implementation. To support transparency, privacy, and expectation management, users should be informed that the prototype does not monitor or do anything until engaged.

To begin a conversation with the robot, the user touches a spot on the robot’s head (a touch button). The robot performs a fixed wakeup routine where it lifts its head, eyes light up, and performs a “stretching” motion by lifting its arms above its head before going to a sitting position. This facilitates anthropomorphism and social engagement and serves as a cue that the robot is ready. It then confirms the touch by asking if the person has time to talk. After finishing the conversation, the robot goes back to a sleeping position, where it lowers its head, and its lights turn off.

#### 3.2.2 Self-reflection conversation design

The premise of our conversation design is for the robot to prompt a user to talk and reflect on their recent thoughts and experiences. As such, the robot asks general questions, to which a user responds, and once the user stops talking, the robot asks general follow up questions. After two follow up questions, the robot asks the user if they would like to continue. While the robot is talking it makes generic hand gestures and uses face tracking to look at the user to support anthropomorphism and increase engagement.

**Figure 2:**
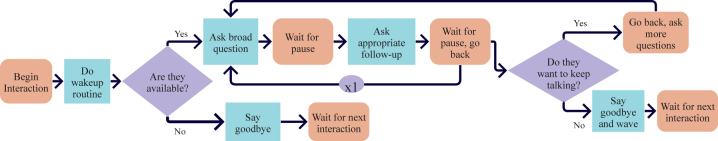
Flow chart of interaction: once a person touches the robot’s head it wakes up, sits (if not already sitting), starts face tracking, and asks the person if they have time to talk. If not, the robot waves goodbye, puts its head down, and returns to its sleeping state (but remains seated). Otherwise, the robot follows the structured conversation where it asks a question, listens until the person is finished, then asks two different follow up questions, waiting each time for them to finish taking. Following, the robot asks if the person wants to keep talking, if they do, it starts another round of 2 questions.

Drawing from self-reflection resources (e.g. [53, 54]), we developed a sample selection of nineteen prompting questions and twenty-five follow up questions. An example prompt is “What has been on your mind lately?” with a potential follow-up of “Okay, tell me about it.” The robot chooses questions and follow ups randomly, with exceptions to ensure the follow-up makes logical sense for a given question. We selected all prompts considering the safety and sensitivity of the questions. We note that further exploration of questions, prompts, and their interaction, as well as the potential for unsafe or harmful utterances, is important to improve the feasibility of the interaction; at this point we aimed for a reasonable selection to enable us to test our simplicity approach. All prompts and follow-ups are given in the Supplementary materials, and a full description and diagram of the interaction protocol is given in Figure 2.

The generic questions and follow ups simplify implementation as the robot does not need to perform speech recognition or semantic analysis. Further, this design does not require deception, and given our design goals of privacy and interaction simplicity (including expectation management) the user should be clearly informed that the robot does not parse utterances or monitor anything.

### 3.3 Implementation

We used a Softbank Nao 25 V6 robot (58 cm tall, Figure 1). Once in a seated position the robot disengages its leg motors to avoid motor wear, vibration, and heat. We used the NaoQi libraries through the Choreographe interface to detect the button press, when the user said “yes” or “no” (as per Figure 2), and perform face following. We further used the NaoQi speech synthesis with the default voice, although we slowed it down to 85% speed based on informal observation to improve understandability of the robot utterances. The robot’s generic talking hand gestures were recorded by an author; we did not use NaoQi’s automated AlAnimatedSpeech class (which generates hand gestures) as they did not work well while the robot was sitting.

To recognize when a person was finished talking, we developed a lightweight algorithm using simple signal processing [61]. Our solution was robust to background noise but could be confused by a loud radio or TV. This technique did not record any sounds that were observed, monitoring microphone amplitude only. We implemented this in Python and integrated it into the NaoQi infrastructure. As a failsafe in case the algorithm did not detect the end of talking, it automatically stopped listening after 4 min.

As all the functionality was processed on the robot, we disabled the robot’s wireless functionality.

## 4 Study – conversational social robot

We designed and conducted a proof-of-concept study to investigate our approach, reflecting on our design goals and the feasibility of interacting with a robot that does not parse utterances. We placed our prototype unsupervised into people’s homes for approximately 48 h (two nights over three different days).

### 4.1 Task

We asked participants to engage the robot to have a conversation whenever and for however long was convenient for them; we did ask participants to aim for at least one conversation per study day given our goal of evaluating interaction.

### 4.2 Instruments and data collection

We used semi-structured interviews at various points in our study to elicit general information such as demographics and participant opinions and attitudes toward robots and technology, as well as details on their experiences with our prototype. We used a standard semi-structured interview approach of a series of guiding questions to focus on specific research investigation goals. All interviews were audio recorded for analysis purposes.

We administered the Almere [62]; acceptance of assistive social robots by older adults) and RoSAS questionnaires [63]; opinions on robot social attributes) to understand general attitudes toward social robots, both at the beginning and end of our study.

The robot recorded general behavior including time of participant interaction, length of interaction, and path through the state machine (Figure 2). At no time was any other information (such as what the person said, video feed, etc.) recorded.

### 4.3 Procedure

We conducted initial consent procedures and study explanation by email. Before noon on the agreed-upon first day we delivered the robot and paper questionnaires in a backpack to the participant.

The researcher conducted training and all interviews virtually due to COVID-19 restrictions. The intake interview started with simple demographics questions and followed with a semi-structured interview on attitudes toward robots and technology. We led participants through unpacking the robot, including practicing turning it on an off, watching it seat itself, and plugging the robot in to charge it; we instructed people to generally leave the robot plugged in, but let them know that it has a battery so they could leave it unplugged for some time. We did not record robot plugging behavior. We further instructed participants that they can place the robot anywhere they wish in their home including on the floor or on a table. We asked the participants to fill the intake copy of the Almere and RoSAS questionnaires.

The researcher explained the task, leading the participant through a full conversation session. To manage expectations, we emphasized that the robot was not intelligent and only followed a simple script. The researcher emphasized that the participant should feel free to turn the robot on and off, or relocate it, as they wished, that it would not stand up, and that the participant was not responsible for any damage to the robot. To develop a sense of privacy we explained that the robot did not record or parse anything the person said (only how loud they were talking), no cameras were enabled, and the robot was not connected to the internet.

We followed with a semi-structured interview focusing on participant initial reactions to and expectations of the robot prototype.

We left the robot with the participant for two nights (over three different days, approximately 48 h), conducting the exit session on the third day before retrieving the robot. Participants again completed the Almere and RoSAS questionnaires, and we conducted a semi-structured interview targeting basic use (e.g., did they use it, how comfortable they felt, robot errors), opinions on the design (e.g., the behavior, the robot’s appearance, how the robot fit into their homes), and potential for long term use (e.g., could they see themselves using something like this for longer, how to make it better).

This study was approved by the university’s ethics board. All participants were compensated with $50 CAD ($25 at the beginning, $25 at the end). We complied with all government and institutional COVID-19 safety protocols, including wearing a mask and gloves during delivery and pickup, and a sanitation routine.

### 4.4 Participants and recruitment

We targeted older adults as they are more likely than younger adults to live alone and experience loneliness [23, 24], and because a narrow demographic would reduce inter-participant variability. We did not specifically target people who identify as lonely at this early stage because we need to determine basic interaction feasibility before engaging with long-term well-being outcomes.

We recruited through social media including our local subreddit in Winnipeg, Manitoba (r/manitoba) and our University’s Centre on Aging network (twitter, email newsletters, Facebook). We noted our inclusion criteria as adults aged 65 and older, who lived alone within city limits and were capable of independently managing a 5 kg robot.

### 4.5 Analysis strategy

We transcribed all interviews and conducted inductive, iterative open coding to extract dominant themes. All transcription and coding were done by the first author; we did not recruit additional coders or do reliability testing given the exploratory study purpose. Our analysis focal points were to reflect on our design goals and the feasibility of our design for self-reflection.

We analyzed our questionnaires (Almere, RoSAS, before study vs. after) using paired, two-tailed *t* tests, to gain insight on the impact of interaction with our prototype on participant attitudes.

## 5 Results

We conducted the study in July and August of 2021. Our recruitment resulted in 14 participants from the Winnipeg (Canada) area aged 66–88 years old (*m* = 71.5, SD = 6.7); 2 were male. All participants lived alone in detached homes or individual apartment units; none lived in group homes or retirement centers, etc. No participant had prior experience with robots. All participants completed the 48 h without requiring researcher intervention.

Interaction logs indicated that the participants had a median of 7 interaction sessions with the robot (max = 28, min = 4, IQR = 2.75). The grand mean session length across participants was 3 m 36 s (min 1:55, max 5:26, SD 1:16).

We found a small decrease in anxiety toward robots during the study (−0.55 on a 6-point scale, SD = 0.53, *t*_13_ = 3.9, *p* = 0.002, *d* = 1.04). We also found a series of negative outcomes including a more negative attitude about robots (−0.33, SD = 0.53, *t*_13_ = 2.4, *p* = 0.034, *d* = 0.63), a lower intention to use for long periods of time (−1.17, SD = 1.7, *t*_13_ = 2.6, *p* = 0.34, *d* = 0.68), a lower sense of robots having a social presence (−0.57, SD = 0.79, *t*_13_ = 2.7, *p* = 0.018, *d* = 0.7), and a lower trust in robots giving advice (−1, SD 1.43, *t*_13_ = 2.6, *p* = 0.021, *d* = 0.70). Participants also reported increased discomfort (1.39 on the 7-point ROSAS scale, SD = 1.27 *t*_13_ = −4.1 *p* = 0.001, *d* = 1.09).

In the remainder of the section, we present the resulting predominant themes (as subsections) and sub-themes (italicized) from our qualitative analysis, based on our interviews with participants.

### 5.1 Potential for reflective conversation

These results focus on the feasibility of the prototype design for self-reflection, reflecting on the underlying design goals intended to facilitate interaction and promote self-reflection behavior. In terms of the robot’s *social engagement*, most participants positively noted the lifelike nature of the robot, e.g., focusing on its humanoid features and movements:

…the gestures it would make with its hands. It was pretty appropriate. The way it asked the question it was inviting – P1, exit.

For example, one participant praised the simplistic humanoid form:

Moving eyes and mouth would have actually been dissonant … would have gotten in a way of me understanding what this robot can do. It would have been a false front. It matches my expectations – P3, exit.

Further, while there were few comments on the robot’s voice, one participant found it problematic:

If only it didn’t talk like Alvin (chipmunk) … one of the reasons I felt so silly was because of its voice … it’s just very annoying – P9, exit.

There were no comments on inappropriate or awkward behaviors or actions such as gaze, hand motions, or other elements of interactive design. Another dominant theme relating to the potential for reflective conversation was *privacy*. Participants commonly discussed the importance of the fact that the conversation was not recorded:

I found this a very safe place to [talk], if I said something that didn’t come out right, I just kept talking because I knew it wasn’t being recorded. It didn’t matter what I said, as long as it was helpful – P5, exit.

I didn’t have anxiety because I knew I wasn’t being recorded – P3, exit.

And particularly that the conversation was not transmitted anywhere:

I’m in the safety and security of my home, I got this little cute guy in front of me who’s listening, and I know it’s not going to go anywhere … I think I would be more hesitant to share otherwise – P6, exit.

Some indicated they were open to recording or transmission, but noted the importance of control and transparency:

If I had some sort of written guarantee that said … the information is only going to here … and have the ability to say a keyword so it would switch the internet and then when you just really want to talk, there’s no internet connection – P4, exit.

And two others noted the potential to transmit to a healthcare provider or to integrate into therapy:

I wouldn’t take advice from a robot, but if they sent [the recording] to a real therapist who would write down any recommendations and then send it back to me, I might take that advice … I think I would feel more comfortable talking to a robot because I know that it hasn’t got any feelings and no memories, so I could say anything I want – P11, exit.

So while some participants did seem to be open to some recording with control and for special circumstances, even so, 11 (out of 14) participants mentioned that recording would have negative impacts on how they engaged the robot:

I think you would be more self-conscious. You’d be more aware. You would have to know how that data was going to be managed – P3, exit.

In terms of the actual *self-reflection* behavior, all participants reported engaging in the activity, and further, that they reflected on the experience. It was common for participants to talk in terms of their own reflection and the activity, and not that the robot itself was an actual social companion:

The prompt takes you out of your head. You’re listening to yourself talk, that’s kind of interesting. It’s not that you think you’re in the presence of an entity that is conscious, but it is a kind of marker that lets you hear yourself in a different way. You become more conscious, but you don’t become self-conscious – P3, exit.

What I found was that verbalizing, like honest, real answers to his questions was very useful – P6, exit.

Perhaps relating to the privacy and engagement points above, participants reported feeling free to talk openly with the robot:

I was very, very open and because I wanted to make the most of this … they are pretty important and useful questions to reflect on – P6, exit.

At the very beginning when it asked a question, I was not very forthcoming in what I answered, but the more that we chatted, the more forthcoming I became – P12, exit.

Despite the generic nature of the questions, participants generally found them to enable of the self-reflection activity:

Most often, the question that came next fit. It seemed to be intuitive to what we’ve just been talking about. Other times not so … but most times, the question lead you on somewhere – P10, exit.

In part due to being socially engaging and successfully creating a sense of privacy, the behavior design was successful in supporting participants to engage in the self-reflection activity.

### 5.2 General prototype design feasibility

Beyond the core goal of engaging participants in self-reflective behavior, we investigated the general feasibility of the overall prototype design in terms of actual use in people’s homes. In terms of *ease of use*, all participants noted the simplicity of interaction:

Because you had very few moves you had to make, that’s very helpful – P3, exit.

All I have to train myself to do is wait for the beep but otherwise I think it’s pretty simple – P1, intake.

And many reported how this supported participant comfort and helped them be confident in using the robot:

I found it very easy to use, I wasn’t apprehensive about using it at all because I was very confident that it was going to go right because of the limited first knowledge I needed – P5, exit.

Further, most participants commented positively on the fact that we explained just how simple the robot was, and through this we helped to manage their expectations:

If I had higher expectations, it probably would have affected me, but after your explanation on the first day, I realized, okay, it’s not going to be understanding what I’m saying anyway, so I didn’t have expectations – P9, exit.

I pretty much knew that it was limited conversations, so I really didn’t have high expectations. I’m not gonna be disappointed – P12, intake.

In terms of the *robot misfunctioning or making errors*, the primary problems noted by participants related to the robot’s conversational timing, for example, interrupting the person or not recognizing when the person stopped talking:

Sometimes I thought it was going to have a problem because it seemed like a longer pause than I was expecting, but then it would start to ask question again. Like I didn’t have any issues at all – P5 exit.

There were a couple of times were there were dead spots … it didn’t make me feel anything, really and truly, when it did kick back in again, we just went merrily on our way – P2, exit.

However, as demonstrated above participants noted how easy they found it to accommodate these situations. Two participants experienced serious errors that required them to reset the robot:

I had to shut it down to get it going again … it happened four times … it wasn’t every time it was every second or third time that it would do that. But I still had seven conversations with it – P12, exit.

But again, this did not restrict the use. One participant talked about how they did not take personal blame for the error:

I did not think that I did anything wrong because I hadn’t manipulated the robot … I certainly didn’t condemn myself because it wasn’t working. I just wanted to see, ‘could I get it to work again’. She is so cute, how could I possibly condemn her. She’s a lively entity – P3, exit.

And in the end, used anthropomorphism to downplay the error and to rationalize the situation. Finally, in terms of *meshing into home environments* all participants readily selected a location for the robot in their homes:

I’m going to put it on the little side table beside the couch where I usually sit and when I want to talk to it, I’ll just turn off the TV and have a little conversation – P8, intake.

It’s staying right here in my den. And I’ll be here every morning and talk to it ‘cause my computer is right here – P12, intake.

As above, participants often explained their choice around planning the space and situation around a time to have conversations. Participants further noted that the robot was not intrusive:

It was not intrusive at all. I mean, it just sat there on the table until I want to have a session – P9, exit.

And most reported being comfortable with leaving the robot turned on, while some turned it off at night. Many commented on the robot’s size, but opinions varied without general agreement:

If it was really small, it wouldn’t be the same, this is just kind of the perfect size. It’s easy to sit on a counter. It’s not intimidating, and I think it’s just really the right size – P4, exit.

I’d have him smaller. I have a small house … he takes up a quarter of my kitchen table, he’s just kinda too big and heavy I think – P10 exit.

No one reported that they thought the robot should be larger. As such, overall participants readily found a place for the robot in their homes, and found the robot easy to use despite the occasional error, which they easily worked around. This provides support for the general interaction and home-integration feasibility of our prototype design.

### 5.3 Potential benefits of prototype

While the above themes focused on interaction and integration feasibility of the overall robot design, in this section we report on potential benefits of the companion robot design. Nine of the 14 participants explicitly noted that they felt specific *self-reflection benefits* of the prototype:

I let out some of the things I bottle up every once in a while. So, from that perspective, it was very useful – P2, exit.

I found that in one instance I had been thinking about it quite a bit and at the point that I sat down to have a chat I said what I had to say and then I didn’t think about it as much afterwards. So, if there’s a psychological perspective to it. For me, that worked, I would never have thought that, but it worked – P5, exit.

If I got a phone call and I was not happy with the phone call or when I was in a meeting yesterday and I was really frustrated afterwards, I could go and then say to it well, Geez, look what this person did and it was a stress reliever for me – P13, exit.

As highlighted in the above comments, participants regularly reported on actual instances where the self-reflection robot served to be useful to them specifically. Even participants who were less positive or noted reservations thought that the prototype may have potential for self-reflection:

I maybe shouldn’t dismiss the role that the robot played, in that I’m actually talking to somebody or something. That might have made a difference. I honestly don’t know. I think it probably did, actually … Clearly having the robot there pushed me to think about these things and behave in a way that I wouldn’t have ordinarily – P6, exit.

Some participants clearly stated that they felt that the robot was not useful to them:

I was playing along; I don’t think it would have been useful to me. I do a lot of reflection anyway … I don’t need someone to do the reflection with – P1, exit.

Beyond the specific usefulness of self-reflection, there was a theme of discussing indirect, *secondary benefits* to having and interacting with the robot. For example, some felt the robot helped with loneliness:

It’s good company. My daughter says I’m antisocial. I like to be alone, but I turn the robot on. I don’t feel so lonely – P11, exit.

All participants noted that the robot served as a social catalyst:

They [neighbors] were very curious what it was like and they kind of giggled and I took a video of it getting itself up and they thought that was quite fascinating. My grandchildren were very fascinated too, they wanted to learn about it, I faced time them about and showed them how the robot was acting – P1, exit.

Above and beyond any potential benefit for themselves, one theme was that it was common for participants to discuss the potential for the prototype to be *useful for others*:

It’s amazing, like to me it just makes so much sense … I keep thinking about people I know that are older, isolated, how easy this could be for them, I think just to have something, other than yelling to the tv ‘cause you don’t agree. It’s like having another person there – P4, exit.

For somebody that’s housebound miss being able to converse with somebody. It might be worthwhile to have this to just pour their heart out to. Even though there it’s not going to solve any problems, but sometimes just talking about it makes a world of difference – P9, exit.

Something like this would be, you know, who are not allowed to have pets, who can’t have things like that. At least this would be something to give them some kind of a connection, and it could literally save someone’s life – P8, exit.

Or useful for a future version of themselves whom may be more isolated:

…if I was living alone, couldn’t get out of the house. Only had contact with people virtually then having a robot there to converse with, probably would be a good idea – P13, exit.

In short, participants generally reported feeling either a direct benefit from their own interaction, or, a potential usefulness for others; we note however that without longitudinal study we cannot reflect on actual benefits

### 5.4 Prototype limitations

Participants reported a range of limitations that they noticed with the specific prototype or the general approach of the robot. For example, participants commonly noted the generic nature of the questions:

The questions they’re obviously preprogrammed and they just don’t seem to have any common theme – P9, exit.

And all participants noted the limited size of the question bank:

Very possibly I’d use it more often (in the future) with a larger question bank – P2, exit.

One common suggestion was to have themes that participants could choose from, based on interest:

I would like to be able to sit down and say talk about family, and questions would come up on it. That would be more useful – P12, exit.

Maybe he could ask questions about physical activity, or nutrition, sleep, getting out for walks … inquiring about some of those other life domains might help people – P6, exit.

We received very little feedback on interaction mechanics. One participant (P1) noted that it would be useful if the robot could repeat itself when it was difficult to understand. Finally, some participants suggested potential additional features beyond the reflective conversation:

They can operate as reminders to do things, ‘did you take your medicine?’ I think that’s a huge issue for aging people – P3, exit.

Capable of telling me go comb your hair or you’ve got an appointment at so and so time. So and so is coming over in a little while. Absolutely remind me to eat. Remind me to go grocery shopping – P14, intake.

It could contact people 3/4 times a day, and if there was no response that they had a phone number to call – P13, exit.

Another common theme was to develop more in-depth, complex forms of the conversation:

It’s a toy right now. I would just consider it a toy like a talking doll. Because it doesn’t have the intelligence to actually interact – P8, exit

Including the robot having some form of emotional intelligence or being able to relate to the person:

The robot is great, it’s unjudgmental, but also unfeeling, you’d want something you could feel was actually, really, following up – P7, exit.

it would not matter what I said to it … there’s no interaction or thought process behind, it’s just recorded answers. There can’t possibly be emotion or thought involved – P8, exit.

Overall, the primary complaints with the robot related to the desire for more features or more complex forms of interaction or support, and not necessarily failures with the specific design as deployed.

## 6 Discussion

The results indicate the success of our design in enabling a robot to facilitate a person to engage in private, reflective conversation at home. Further, the results highlight the success of the design approach of targeting interactive behavior without requiring the robot to understand what the person is saying. However, the primarily limitation with our work is that without longitudinal inquiry we do not know whether these interactions would persist, or would actually lead to tangible benefits such as reduced effects of loneliness; a key goal of future work will be to explore the trajectory of interaction over time, looking for pinch points and for ways to support sustained use. Below we discuss the primary findings and limitations of our work.

### 6.1 Reflective-listening design

The results indicate the success of our self-reflection interaction template, as all participants readily understood and engaged with the task; we found no evidence of resistance or confusion with the interaction or behavior itself. That is, despite the fact that the robot did not parse what participants said, people were still able to engage with self-reflection with the robot. We note that although being instructed to use the robot at least once a day, participants had a median of 7 sessions over the 48 h, indicating willingness and ability to engage. There is even indication that the reflection may support well-being, as some noted that they felt the experience was beneficial even during the short deployment.

### 6.2 Social engagement

The anthropomorphic design successfully facilitated our desired interaction, as people reported feeling comfortable talking with the robot and noted the appropriateness of the movements and gestures. Apart from one participant who found the voice “silly,” no participants reported a negative experience such as finding interaction contrived or awkward.

### 6.3 Interaction simplicity

All participants praised the simplicity of operating the robot despite all having no prior experience with robots. Thus, our simplicity approach was a success in that robot operation did not detract from the core task of self-reflection. Further, participants noted that they appreciated the explanation of the robot’s simplicity; this matches previous results where people have better perceptions of robots when their expectations are lowered [64].

### 6.4 Safety and privacy

People reported feeling comfortable talking candidly with the robot, indicating the success of our safety and privacy design. Further, participants noted the importance of the privacy per se, providing additional support for privacy as a key issue for companion robots. Participants particularly noted the implementation transparency (that we told them how it worked), and that the robot was offline.

### 6.5 Implementation simplicity

Our prototype was sufficiently robust to be deployed, unattended and unmonitored, and with minimal user training, for 48 h into the homes of 14 participants. Although some technical problems did occur, these did not hinder interaction and participants were able to recover in all cases. This provides positive support for our general approach of aiming to reduce technical or sensing needs through interaction design.

A key participant criticism of the robot prototype was the lack of depth of interaction, including concerns over the generic nature of the questions and the limited size of the test bank, with participants suggesting advancements such as more intelligence, that our design approach was aiming to avoid. This draws into question the scalability of the approach to more in-depth interactions, as well as the sustainability of interaction over the longer term, highlighting the need for longitudinal study of interaction with our prototype.

Further, after participating in our study participants developed reduced opinions on the potential for robots in general, including a more negative general attitude, lower trust in robot advice, more discomfort, and a lower intention to use a robot for long periods of time. This contrasts with the generally positive interview results, which provided support for the plausibility of the prototype for self-reflection and unmonitored deployment into homes, and that involvement in our study may have reduced participant anxiety toward robots. One explanation is that perhaps expectations were artificially high (e.g., based on media) and our study simply reduced expectations to be more realistic. For example, participants suggested a range of additional features unrelated to the task, such as detecting when one should comb their hair. Finally, participants noted a range of potential uses for other people, even when they did not see a use for themselves. This is a pattern established in the literature, which may be a sign of participants simply being more polite and optimistic in interviews (in comparison to questionnaires) despite not intending to use a device themselves (e.g., see [22, 65]). However, despite this negativity we note that our primary goals – of enabling self-reflection with a standalone robot – were met; even if participants were disappointed and had their opinions lowered through the study, they were still able to have self-reflection sessions with a fully autonomous robot, which may potentially provide benefits.

Overall, these results highlight the potential for our self-reflection interaction template to be used in human-robot interaction, either as is, or as an inspiration for similar interactions or approaches. The combination of our simple interaction design and focus on security and privacy, while taking direction from self-reflection psychology, culminated in a package that enabled people to comfortably reflect on sensitive topics with a robot without requiring advanced robot intelligence. Further, participants readily integrated the prototype into their homes and daily lives, a key challenge of technology adoption [66]. Finally, our technical approach resulted in a robust solution that could be deployed without requiring monitoring, a remote operator, or intense processing that requires online architecture. This lack of monitoring and improved ecological validity greatly increases the validity of our results [47].

### 6.6 Limitations and future work

The key limitation of our work is our inability to reflect deeply on the prototype’s impact on participant well-being, in part because of the short 48 h deployment. Second, while we targeted a demographic which is statistically more likely to be lonely (older adults), as we did not target individuals who said they were lonely or desired improved well-being (to simplify our recruitment) it can be difficult to extrapolate to real results with this population. Finally, our current study design does not involve a required baseline condition: for a longer-term deployment we would require a parallel condition which does not involve the robot, but still involves interaction with the researcher. This way, if we found that people became less lonely over time in our robot condition but not in the no-robot condition, we could more confidently reflect on the impact of our prototype while factoring out participant engagement with the research team.

The short term deployment further means that novelty is a large factor in participant reactions, making it difficult to extrapolate our findings to how participants may behave over time. For example, the robot may not serve as such a social catalyst when it is no longer new and interesting within a social group, and longitudinal study is required to better understand how interaction will evolve. However, we do not see novelty as a study confound per se and emphasize the importance that novelty plays in technology adoption (e.g., as discussed in [67]); understanding how novelty impacts initial use, and how this changes over time, will be important for designing successful conversational robots. We envision that the overall simplicity will support prolonged adoption, in part by avoiding abandonment influenced by complexity [22], but further study is required to better develop our understanding of adoption patterns for companion robots.

Our work was limited by the participant demographic, as our sample was primarily female older adults. While this follows a recorded pattern of older women being more likely to volunteer for such studies [22, 25]; perhaps due in part to the fact that women live longer and are less likely to remarry than men [68], it indicates a need for future work to actively target a balanced sample.

Finally, there remain complex underlying concerns regarding the ethics of these social robots, and ongoing work needs to address the pros and cons of using a social robot as a potential proxy for human interaction (in this case, possibly a health care worker). Particularly in this case where people may potentially disclose serious information that the robot may dismiss, whereas a human could act such as by seeking emergency help, the nuances of choosing such technologies need to be more fully considered. That said, our robot design aims to avoid these issues by presenting the robot as more of an appliance or tool akin to a diary, than a social companion, which helps to alleviate some of these concerns.

## 7 Conclusions

This paper presents a novel conversational companion robot design that does not parse or interpret user speech, and yet still enables successful interaction and facilitates self-reflection. Further, early indicators are that this design could potentially support well-being. Our design-oriented approach successfully triangulated a range of goals, including self-reflection facilitation and social engagement, simple interaction, privacy and security, all with an implementation simplicity that enabled fully autonomous, in-home deployment for ecologically-valid study. Our exploratory qualitative analysis provides encouraging support for our design approach, and the potential our approach (and our specific prototype) for longer term use. We envision that our prototype, design approach, and results, will help direct ongoing development for feasible conversational social robot behaviors.

## Supplementary Material

Supplementary Material Details

## References

[1] Young J. E., Sung J., Voida A., Sharlin E., Igarashi T., Christensen H. I., Grinter R. E. (2011). Evaluating human-robot interaction: focusing on the holistic interaction experience. Int. J. Soc. Robot..

[2] Banks M. R., Willoughby L. M., Banks W. A. (2008). Animal-assisted therapy and loneliness in nursing homes: use of robotic versus living dogs. J. Am. Med. Dir. Assoc..

[3] Aminuddin R., Sharkey A., Levita L. (2016). Interaction with the Paro robot may reduce psychophysiological stress responses. 2016 11th ACM/IEEE International Conference on Human-Robot Interaction (HRI).

[4] Björling E. A., Rose E., Davidson A., Ren R., Wong D. (2020). Can we keep him forever? Teens’ engagement and desire for emotional connection with a social robot. Int. J. Soc. Robot..

[5] Feil-Seifer D., Matarić M. J. (2005). Defining socially assistive robotics. Proceedings of the 2005 IEEE 9th International Conference on Rehabilitation Robotics, 2005.

[6] Lee K. M., Jung Y., Kim J., Kim S. R. (2006). Are physically embodied social agents better than disembodied social agents? The effects of physical embodiment, tactile interaction, and people’s loneliness in human–robot interaction. Int. J. Hum. Comput. Stud..

[7] Nishio T., Yoshikawa Y., Sakai K., Iio T., Chiba M., Asami T., Isoda Y., Ishiguro H. (2021). The effects of physically embodied multiple conversation robots on the elderly. Front. Robot. AI.

[8] Suganuma S., Sakamoto D., Shimoyama H. (2018). An embodied conversational agent for unguided internet-based cognitive behavior therapy in preventative mental health: feasibility and acceptability pilot trial. JMIR Ment. Health.

[9] Pennebaker J. W., Chung C. K. (2012). Expressive writing: connections to physical and mental health. Oxford Handbook of Health Psychology.

[10] Wilson T. D., Gilbert D. T. (2008). Explaining away: a model of affective adaptation. Perspect. Psychol. Sci..

[11] Hickson H. (2011). Critical reflection: reflecting on learning to be reflective. Reflective Pract..

[12] Lin X., Hmelo C., Kinzer C. K., Secules T. J. (1999). Designing technology to support reflection. Educ. Technol. Res. Dev..

[13] Lepore S. J. (1997). Expressive writing moderates the relation between intrusive thoughts and depressive symptoms. J. Pers. Soc. Psychol..

[14] Hicks A. M., Diamond L. M. (2008). How was your day? Couples’ affect when telling and hearing daily events. Pers. Relat..

[15] Seikkula J., Trimble D. (2005). Healing elements of therapeutic conversation: dialogue as an embodiment of love. Fam. Process.

[16] Chopik W. J. (2016). The benefits of social technology use among older adults are mediated by reduced loneliness. Cyberpsychol., Behav. Soc. Netw..

[17] Brandtzaeg P. B., Følstad A. (2017). Why people use chatbots. Internet Science. INSCI 2017. Lecture Notes in Computer Science.

[18] Sarabia M., Young N., Canavan K., Edginton T., Demiris Y., Vizcaychipi M. P. (2018). Assistive robotic technology to combat social isolation in acute hospital settings. Int. J. Soc. Robot..

[19] Hoffman G. (2019). Anki, Jibo, and Kuri: what we can learn from social robots that didn’t make it. IEEE Spectrum Magazine.

[20] Potts C., Bond R., Mulvenna M. D., Ennis E., Bickerdike A., Coughlan E. K., Broderick T., Burns C., McTear M. F., Kuosmanen L., Nieminen H., Boyd K. A., Cahill B., Vakaloudis A., Dhanapala I., Vartiainen A. K., Kostenius C., Malcolm M. (2021). Insights and lessons learned from trialling a mental health chatbot in the wild. 2021 IEEE Symposium on Computers and Communications (ISCC).

[21] Fiske A., Henningsen P., Buyx A. (2020). The implications of embodied artificial intelligence in mental healthcare for digital wellbeing. Ethics of Digital Well-Being. Philosophical Studies Series.

[22] Frennert S., Eftring H., Östlund B. (2017). Case report: implications of doing research on socially assistive robots in real homes. Int. J. Soc. Robot..

[23] Coyle C. E., Dugan E. (2012). Social isolation, loneliness and health among older adults. J. Aging Health.

[24] Tang J., Galbraith N., Truong J. (2019). Living alone in Canada.

[25] Abdi J., Al-Hindawi A., Ng T., Vizcaychipi M. P. (2018). Scoping review on the use of socially assistive robot technology in elderly care. BMJ Open.

[26] Kachouie R., Sedighadeli S., Khosla R., Chu M. T. (2014). Socially assistive robots in elderly care: a mixed-method systematic literature review. Int. J. Hum. Comput. Interact..

[27] Whelan S., Murphy K., Barrett E., Krusche C., Santorelli A., Casey D. (2018). Factors affecting the acceptability of social robots by older adults including people with dementia or cognitive impairment: a literature review. Int. J. Soc. Robot..

[28] Carros F., Meurer J., Löffler D., Unbehaun D., Matthies S., Koch I., Wieching R., Randall D., Hassenzahl M., Wulf V. (2020). Exploring human-robot interaction with the elderly. Proceedings of the 2020 CHI Conference on Human Factors in Computing Systems.

[29] Carros F., Schwaninger I., Preussner A., Randall D., Wieching R., Fitzpatrick G., Wulf V. (2022). Care workers making use of robots: results of a three-month study on human-robot interaction within a care home. CHI Conference on Human Factors in Computing Systems.

[30] Sabelli a. M., Kanda T., Hagita N. (2011). A conversational robot in an elderly care center: an ethnographic study. Human-Robot Interaction (HRI), 2011 6th ACM/IEEE International Conference On.

[31] Chen S., Jones C., Moyle W. (2018). Social robots for depression in older adults: a systematic review. J. Nurs. Scholarsh..

[32] McGlynn S. A., Geiskkovitch D., Mitzner T. L., Rogers W. A. (2016). PARO’s stress-reduction potential for older adults. Proceedings of the Human Factors and Ergonomics Society.

[33] Weizenbaum J. (1983). ELIZA—a computer program for the study of natural language communication between man and machine. Commun. ACM.

[34] Abd-Alrazaq A. A., Rababeh A., Alajlani M., Bewick B. M., Househ M. (2020). Effectiveness and safety of using chatbots to improve mental health: systematic review and meta-analysis. J. Med. Internet Res..

[35] Fitzpatrick K. K., Darcy A., Vierhile M. (2017). Delivering cognitive behavior therapy to young adults with symptoms of depression and anxiety using a fully automated conversational agent (woebot): a randomized controlled trial. JMIR Ment. Health.

[36] Hirano M., Ogura K., Kitahara M., Sakamoto D., Shimoyama H. (2017). Designing behavioral self-regulation application for preventive personal mental healthcare. Health Psychol. Open.

[37] Jeong S., Aymerich-Franch L., Arias K., Alghowinem S., Lapedriza A., Picard R., Park H. W., Breazeal C. (2022). Deploying a robotic positive psychology coach to improve college students’ psychological well-being. User Model. User-Adapted Interact..

[38] Luo B., Lau R. Y. K., Li C., Si Y. (2022). A critical review of state‐of‐the‐art chatbot designs and applications. *WIREs Data Min. Knowl. Discov.*.

[39] Nakagawa K., Shiomi M., Shinozawa K., Matsumura R., Ishiguro H., Hagita N. (2013). Effect of robot’s whispering behavior on people’s motivation. Int. J. Soc. Robot..

[40] Shum H., He X., Li D. (2018). From Eliza to XiaoIce: challenges and opportunities with social chatbots. Front. Inf. Technol. Electron. Eng..

[41] Lee M., Ackermans S., van As N., Chang H., Lucas E., IJsselsteijn W. (2019). Caring for vincent. Proceedings of the 2019 CHI Conference on Human Factors in Computing Systems.

[42] Dino F., Zandie R., Abdollahi H., Schoeder S., Mahoor M. H. (2019). Delivering cognitive behavioral therapy using A conversational social robot. 2019 IEEE/RSJ International Conference on Intelligent Robots and Systems (IROS).

[43] Ostrowski A. K., Breazeal C., Park H. W. (2022). Mixed-method Long-Term Robot Usage: Older Adults’ Lived Experience of Social Robots. 2022 17th ACM/IEEE International Conference on Human-Robot Interaction (HRI).

[44] Papadopoulos C., Castro N., Nigath A., Davidson R., Faulkes N., Menicatti R., Khaliq A. A., Recchiuto C., Battistuzzi L., Randhawa G., Merton L., Kanoria S., Chong N. Y., Kamide H., Hewson D., Sgorbissa A. (2022). The CARESSES randomised controlled trial: exploring the health-related impact of culturally competent artificial intelligence embedded into socially assistive robots and tested in older adult care homes. Int. J. Soc. Robot..

[45] Björling E. A., Ling H., Bhatia S., Dziubinski K. (2020). The experience and effect of adolescent to robot stress disclosure: a mixed-methods exploration. Social Robotics. ICSR 2020. Lecture Notes in Computer Science.

[46] Riek L. (2012). Wizard of Oz studies in HRI: a systematic review and new reporting guidelines. *J. Hum. Robot Interact.*.

[47] McCarney R., Warner J., Iliffe S., van Haselen R., Griffin M., Fisher P. (2007). The Hawthorne Effect: a randomised, controlled trial. BMC Med. Res. Methodol..

[48] Mubin O., Henderson J., Bartneck C. (2014). You just do not understand me! Speech Recognition in Human Robot Interaction. The 23rd IEEE International Symposium on Robot and Human Interactive Communication.

[49] Liu H., Peng H., Song X., Xu C., Zhang M. (2022). Using AI chatbots to provide self-help depression interventions for university students: a randomized trial of effectiveness. Internet Interventions.

[50] van Maris A., Zook N., Caleb-Solly P., Studley M., Winfield A., Dogramadzi S. (2020). Designing ethical social robots—a longitudinal field study with older adults. Front. Robot. AI.

[51] Bickmore T. W., Trinh H., Olafsson S., O’Leary T. K., Asadi R., Rickles N. M., Cruz R. (2018). Patient and consumer safety risks when using conversational assistants for medical information: an observational study of Siri, Alexa, and google assistant. J. Med. Internet Res..

[52] Hutchinson H., Hansen H., Roussel N., Eiderbäck B., Mackay W., Westerlund B., Bederson B. B., Druin A., Plaisant C., Beaudouin-Lafon M., Conversy S., Evans H. (2003). Technology probes. Proceedings of the Conference on Human Factors in Computing Systems – CHI ’03.

[53] Joseph S., Murphy D. (2013). Person-centered approach, positive psychology, and relational helping: building bridges. J. Humanist. Psychol..

[54] Rogers C. R. (1981). The foundations of the person-centered approach. Dialectics Humanism.

[55] Seo S. H., Geiskkovitch D., Nakane M., King C., Young J. E. Poor thing! Would you feel sorry for a simulated robot? A comparison of empathy toward a physical and a simulated robot. Proceedings of the Tenth Annual ACM/IEEE International Conference on Human-Robot Interaction (HRI ’15).

[56] Fischer K., Weigelin H. M., Bodenhagen L. (2018). Increasing trust in human–robot medical interactions: effects of transparency and adaptability. Paladyn. J. Behav. Rob..

[57] Paepcke S., Takayama L. (2010). Judging a bot by its cover: an experiment on expectation setting for personal robots. 5th ACM/IEEE International Conference on Human-Robot Interaction, HRI 2010.

[58] Lee M. K., Tang K. P., Forlizzi J., Kiesler S. (2011). Understanding users’ perception of privacy in human-robot interaction. Proceedings of the 6th International Conference on Human-Robot Interaction – HRI ’11.

[59] González A. L., Geiskkovitch D. Y., Young J. E. (2020). When can I get a robot for my home? A constrained design approach to feasible, deployable companion. Proceedings of the RO-MAN 2020 Workshop on Social Human-Robot Interaction of Human-Care Service Robots. At the 29th International Conference on Robot & Human Interactive Communication (ROMAN 2020).

[60] González A. L., Young J. E. (2020). Please tell me about it. Proceedings of the 8th International Conference on Human-Agent Interaction.

[61] González A. L., Young J. E. (2020). A Simple and Lightweight Algorithm for Social Robot Speech Turn Taking.

[62] Heerink M., Kröse B., Evers V., Wielinga B. (2010). Assessing acceptance of assistive social agent technology by older adults: the almere model. Int. J. Soc. Robot..

[63] Carpinella C. M., Wyman A. B., Perez M. A., Stroessner S. J. (2017). The robotic social attributes scale (RoSAS): development and validation. ACM/IEEE International Conference on Human-Robot Interaction, Part F1271.

[64] Fernaeus Y., Håkansson M., Jacobsson M., Ljungblad S. (2010). How do you play with a robotic toy animal?. Proceedings of the 9th International Conference on Interaction Design and Children – IDC ’10.

[65] Lazar A., Thompson H. J., Piper A. M., Demiris G. (2016). Rethinking the design of robotic pets for older adults. Proceedings of the 2016 ACM Conference on Designing Interactive Systems – DIS ’16.

[66] Young J. E., Hawkins R., Sharlin E., Igarashi T. (2008). Toward acceptable domestic robots: applying insights from social psychology. Int. J. Soc. Robot..

[67] Smedegaard C. V. (2019). Reframing the role of novelty within social HRI: from noise to information. 2019 14th ACM/IEEE International Conference on Human-Robot Interaction (HRI).

[68] Davidson K. (2017). Gender differences in new partnership choices and constraints for older widows and widowers. Intimacy in Later Life.

