# Supplementary material for: Say what you want, I’m not listening! A conversational self-reflection robot that does not parse user speech
Source: I Com (Berl). 2023 Mar 10;22(1):19–32. doi: 10.1515/icom-2022-0047 (PMC10081923; doi:10.1515/icom-2022-0047)
Supplement: Supplementary file 1 — Supplementary Material Details [file j_icom-2022-0047_suppl.docx]

**Appendix A**

Questions and Follow Ups

As explained in the main paper, we had 19 main questions the robot asked and 25 follow up phrases. Our robotic behavior chose at random two main questions and a follow up for each. This appendix shows the questions, and the follow ups for each of the questions.

The 19 main questions asked by the robot were the following:

1. Are you satisfied with what you're doing in your life?
2. Who was the last person you saw?
3. Is there something you want to change in your life?
4. What do your friends mean to you?
5. Is something bothering you?
6. How do you feel about your family?
7. Has anything good or bad happened lately?
8. What has been on your mind lately?
9. How has your life changed in the past year?
10. What would you change if you had the chance?
11. What are you most proud of?
12. What do you like about your life?
13. What do you like doing by yourself or with others?
14. What are you grateful for today?
15. If you could be doing something else, what would that be?
16. What is the last thing someone said to you that made you feel good?
17. What was the last news that made you happy?
18. When was the last time you did something different?
19. What made you happy lately?

The 25 follow up questions or phrases to encourage people to continue talking were the following:

1. Okay tell me about it
2. In what way is this good or bad?
3. Are you happy with this?
4. Are you satisfied with that?
5. How did this end up happening?
6. Is there a reason for this?
7. Do you feel better or worse by talking?
8. Tell me more about this.
9. Is this good? If not, can you change it?
10. ok. please go on.
11. Do you often feel this way?
12. What do you think about that?

1. Where do these feelings come from?
2. What makes you feel this way?
3. How do you feel after talking?
4. Who said that?
5. Why did they say that?
6. What could make you feel better?
7. Would you have it any other way?
8. Do you have any regrets from this?
9. Why do you say that?
10. What happened to make you feel like this?
11. How did you end up here?
12. How did that go?
13. Why did that happen?

We knew not all follow ups were appropriate matches for each of the main questions. Because of this we created a list to match each question with an appropriate follow up. The numbers on the left correspond to the numbers from the main questions and the numbers in brackets correspond to possible follow ups that could be chosen for each main question.

1: [ 1,2, 5,6,7, 9,10,11, 13,14,15, 18,19, 21,22,23, 25 ]

2: [ 1,2,3,4,5,6, 8,9, 12, 19,20, 23,24,25 ] 3: [ 2,3, 6,7,8, 12,13,14, 18,19, 21,22,23, 25 ]

4: [ 1, 3, 6, 8,9,10, 13,14,15, 18,19, 21,22 ]

5: [ 1, 5,6, 8, 10,11,12,13,14,15, 18, 21,22,23, 25 ]

6: [ 2,3,4, 6,7, 9,10, 13,14, 19,20, 22, 25 ]

7: [ 1, 3,4,5,6, 8,9,10,11,12,13,14,15, 18,19,20,21,22,23, 25 ]

8: [ 1,2,3, 6, 8,9,10, 12,13,14, 18,19, 21,22, 25 ]

9: [ 2,3,4,5,6, 8,9, 12, 14,15, 18,19,20,21,22, 25 ]

10: [1,2, 6, 8,9,10,11, 13,14, 21,22,23, 25 ]

11: [ 1, 3,4,5,6, 8, 10, 13,14, 18,19, 21,22, 24,25 ]

12: [ 3,4, 6,7,8, 10, 13,14,15, 18,19, 21,22 ]

13: [ 1, 6, 8, 10, 12, 14, 19, 21,22, 25 ]

14: [ 1,2,3, 6, 8, 10,11,12,13,14, 19, 21,22,23 ]

15: [ 6, 8,9,10,11, 13,14, 21,22,23 ]

16: [ 1, 6, 8, 10, 12,13,14, 16,17,18,19, 21, 24,25 ] 17: [ 1, 5,6,7,8, 12, 14,15, 21,22, 25 ]

18: [ 1,2,3,4,5,6, 8,9,10, 12, 15, 19,20, 24,25 ] 19: [ 1, 4,5,6,7,8, 10,11, 13, 18,19, 21, 24,25 ]
